# Letheon

**Published:** 1847-04

**Authors:** 


ARTICLE XI.

### LETHEON.

The use of the ethereal vapor, or Letheon, has been adopted more rapidly and has been more generally successful than any other important improvement or discovery in medicine of which we have any recollection. But a few months have elapsed since its application was first made public in Boston, and now it is in almost universal use in this country, and the European Journals are full of accounts of its application by our transatlantic brethren of the healing art. It has been used to prevent pain in almost all of the important and minor operations in surgery with entire success, and the experiment has been tried of giving it to prevent the pain of parturition. It is said to succeed admirably in this—relieving the pain or sufferings of the patient without interfering with the contractions of the uterus. In application to patients in labor it has mostly been made for the purpose of applying the forceps, though it has been used in a few cases of natural labor. In one there was produced great cerebral congestion, but as yet no other serious result has arisen from it. M. Dubois, deservedly at the head of this department of medicine in France, thinks it will only be appropriately applied in extreme cases.

Its influence on insane petients has been tried at the Bicetre, by M. Moreau, but with decidedly unfavorable results.

This discovery, which is likely to form an epoch in the history of our profession is claimed, as might be expected, by numerous individuals, both in Europe and this country. But whatever may be the claims of others, it is certain that the attention of the profession and the world, was first successfully attracted to it by Dr. Jackson, of Boston. In some brief remarks upon this subject, made by Dr. Jackson, at a meeting after the close of the recent session of the medical school in Boston, reported in the Boston Med. and Surg. Journal, he said:

"He was aware of the pretensions advanced by others, but he believed that they had not been countenanced by the scientific world. The Academy of Sciences, of France, had received this discovery, and acknowledge its value, and had recorded it as emanating from America. They had set aside at once, the claims of pretenders, and had acted justly and honorably.

"He had already given to the public an account of the original experiments which he had made, on the effects of ether vapor. He would only re-assert, as he can prove by the testimony of others, that he discovered that insensibility to pain was produced by inhalation of sulphuric ether vapor, and that he communicated the fact to one of his pupils in February, 1846, and requested him to try the experiment when he had a tooth extracted.

"In the latter part of September last, he communicated this discovery to a dentist of this city (Mr. W. T. G. Morton), and requested him to administer the ether to one of his patients, with the assurance that it would produce insensibility, and that the experiment would be free from danger, if his directions were followed. He regarded himself as responsible for the results of the first experiments, which were made at his suggestion and by his advice. He next requested that dentist to go to the Massachusetts General Hospital, and ask Dr. Warren's permission to administer the ether vapor to a patient about to undergo a surgical operation.

"He regretted that any misunderstanding should have arisen concerning this discovery. He was willing to allow great enterprise and zeal in promoting its introduction, and improving his originally simple apparatus. He did not see any reason why each party should not be willing to rest content with what they had done.

It would certainly be unwarrantable for the miner, who carried Davy's safety lamp into the fire damps of a mine, to dispute the claims of its original inventor; for he received that instrument already proved to be efficient, with the assurance that it would guide him in safety through the explosive gases of the mine."

